# Sabin inactivated polio vaccine upstream process development using fixed-bed bioreactor technology

**DOI:** 10.1016/j.vaccine.2025.126950

**Published:** 2025-04-19

**Authors:** Ahd Hamidi, Marieke Willemsen, Thomas Robert, Jean-Christophe Drugmand, Mónika Z. Ballmann, Pim Velthof, Hans Verdurmen, Ana Catarina Pinto, Jochem Pronk, Laura Palladino, Menzo Havenga, Chris Yallop, Wilfried A.M. Bakker

**Affiliations:** a Batavia Biosciences B.V., Bioscience Park Leiden, Zernikedreef 16, 2333CL, Leiden, the Netherlands; b Univercells Technologies SA, Chemin De la Vieille Cour 56a, 1400 Nivellles, Belgium

**Keywords:** Equipment development, Fixed-bed bioreactor, Tangential flow filtration, Vaccines, Low cost of goods, Inactivated polio vaccine, sIPV, Sabin attenuated poliovirus

## Abstract

Eradication of polio disease remains a challenge for countries with limited health-care infrastructure. Regional vaccine production is expected to secure a sustainable and equitable availability of vaccines supporting the polio eradication end-game. Regional manufacturing of Inactivated Polio Vaccines based on the attenuated Sabin strains and using an isolator-based contained micro-facility is expected to avoid any potential risk of shortage of polio vaccines in the future, ensure equitable access to sufficient doses of IPV while securing a safe manufacturing environment. However, polio vaccine production requires biosafety level 3 containment and is complicated by the restrictions imposed from the adherent nature of the virus-producing Vero cell line. To overcome these issues that have hampered regional polio vaccine production so far, at Batavia Biosciences we developed an inactivated polio vaccine production process based on the polio Sabin strains in a contained microfacility. By incorporating a tangential flow filter coupled to a fixed-bed bioreactor with a large attachment surface area (150 m^2^), we could increase process efficiency and reduce the production footprint allowing for regional demand-driven production. The reduced square meters of the manufacturing site that handles the live virus were achieved by integrating the fixed-bed bioreactor with the concentration step (TFF). The increased efficiency was realized by using less resources, fully disposable virus production, and a down- and up-scalable process depending on vaccine demand. The here-developed scalable production process with reduced production footprint is considered a useful and cost-effective method for regional vaccine production, for example in pandemic preparedness efforts to efficiently contain virus outbreaks.

## Highlights

• Poliovirus vaccine production is feasible using a low-footprint container-sized production site.
• Structured fixed-bed bioreactors enable linear upscaling of Vero cell growth and virus production.
• A tangential flow filter coupled to a fixed-bed bioreactor increased process efficiency and reduced the production footprint.
• The developed scalable production process is considered a useful and cost-effective method for regional vaccine production.
• Regional isolator-based sIPV manufacturing is expected to ensure equitable access to sufficient doses of IPV.

## 1 Introduction

Poliomyelitis (polio) is an infectious disease caused by non-enveloped poliovirus (PV) with a single-stranded RNA viral genome. Polio is considered a suitable target for eradication through an all-encompassing vaccination campaign. However, current vaccine manufacturing costs and limited availability of the vaccines still pose an obstacle to the successful eradication of polio disease, especially in resource-limited countries [1,2]. The Global Polio Eradication Initiative (GPEI) and the World Health Organization (WHO) Strategic Advisory Group of Experts recommend the cessation of conventional, relatively cheap live oral polio vaccine (OPV), as this vaccine can lead to rare cases of vaccine-associated paralytic polio and circulating vaccine-derived polioviruses [2]. As an alternative, inactivated polio vaccines (IPV) are nowadays produced using attenuated Sabin poliovirus strains. Inactivation of the polio virus during manufacturing will prevent vaccine-associated paralytic polio and circulating vaccine-derived poliovirus cases [2]. Attenuations of the viral genome have reduced the viral neurovirulence, but mutations during viral propagation can revert the reduction of neurovirulence. Therefore, in-process controls during viral propagation in the commercial manufacturing process need to be established to control low mutations of the viral genome.

Due to its efficacy in preventing paralysis, Inactivated Polio Vaccine (IPV) was the vaccine of choice in high-income settings, administered as a three-dose regimen often including one or two booster doses [2]. It was only in 2019 that GAVI-supported countries introduced the first dose of IPV, the switch from a one-dose schedule to a two-dose schedule is progressing since 2021. Starting from 2024, GAVI supports gradual access to the hexavalent vaccine (DTP-HepB-Hib-IPV) making it necessary to use four doses of IPV [3]. GPEI advises all countries to use at least two IPV doses for at least 10 years after cessation of the last OPV serotype and suggests this as a prerequisite to the ultimate cessation of all OPV use in routine immunization (World Health Organization, 2017). To ensure sufficient vaccine supply, the GPEI additionally aims to accelerate the development and use of novel genetically stabilized hyper-attenuated strains (nOPVs, with less risk for neurovirulence reversion) and attenuated Sabin IPV (sIPV) vaccines (WHO Polio Eradication Strategy 2022–2026) [4]. Vaccine manufacturing typically requires large-scale facilities to utilize an economy-of-scale strategy to achieve an attractive commercial unit price [5,6], which are however capital intensive. In contrast, we have developed a scalable sIPV manufacturing upstream process that allows small-scale, small manufacturing footprint, regional, and contained virus production.

Production costs of IPVs compared to OPVs are generally higher due to the need for purification and additional product losses during virus inactivation [7]. While this inactivation step cannot be omitted, one major process efficiency limitation stems from the fact that polio vaccine production is performed almost exclusively using the WHO Vero 10–87 cell line which needs an attachment surface for adherent cell growth. This adherent nature of the WHO Vero 10–87 cell line, however, complicates the scale-up and intensification of the cell growth process [8]. The current manufacturing standard to produce IPVs uses the WHO Vero 10–87 cell line cultured on flatware or microcarriers [9,10] with subsequent infection by attenuated Sabin strains. Following viral infection, the obtained virus is harvested, clarified, purified, and inactivated [9,11].

Microcarrier-based manufacturing provides a scale-up of the cell culture process using well-defined suspension process technology, however, a Vero cell-based micro-carrier process requires a large manufacturing footprint as the cell density of the cell culture is usually low. In general, agitation needs to be minimized for Vero cell growth to minimize shear stress, and aeration is often only performed via the bioreactor headspace which has a poor volumetric mass transfer coefficient [12,13].

In this paper, we present a newly developed compact sIPV production process based on a novel fixed-bed bioreactor design that follows the safety-by-design principles [14]. The scale-X™ bioreactor uses a structured, spiral-wound fixed-bed matrix to offer a controlled and reproducible environment. This design has been shown to drive increased specific productivities in various applications [15,16]. The present study details the transfer and scale-up of the sIPV production process into this fixed-bed bioreactor.

We characterize Vero cell growth and sPV production at 2.4 m^2^ surface area bioreactor scale and show that scale-up of the technology to 30 m^2^ is feasible, with a first proof-of-concept performed at 150 m^2^ manufacturing scale. The 150 m^2^ scale sIPV production process was enclosed into a designed-for-purpose contained isolator-based microfacility prototype, enabling continuous processing and facilitating compliance with WHO Global Action Plan (GAP) for Poliovirus Containment biosecurity requirements to prevent viruses from escaping into the environment or exposure of operators [4].

## 2 Materials & methods

All activities at Batavia Biosciences with live polio virus were performed in research laboratories under BSL3 conditions, in line with applicable regulations at the time that this work was performed.

### 2.1 Structured fixed-bed bioreactor design

**Fig. 1:**
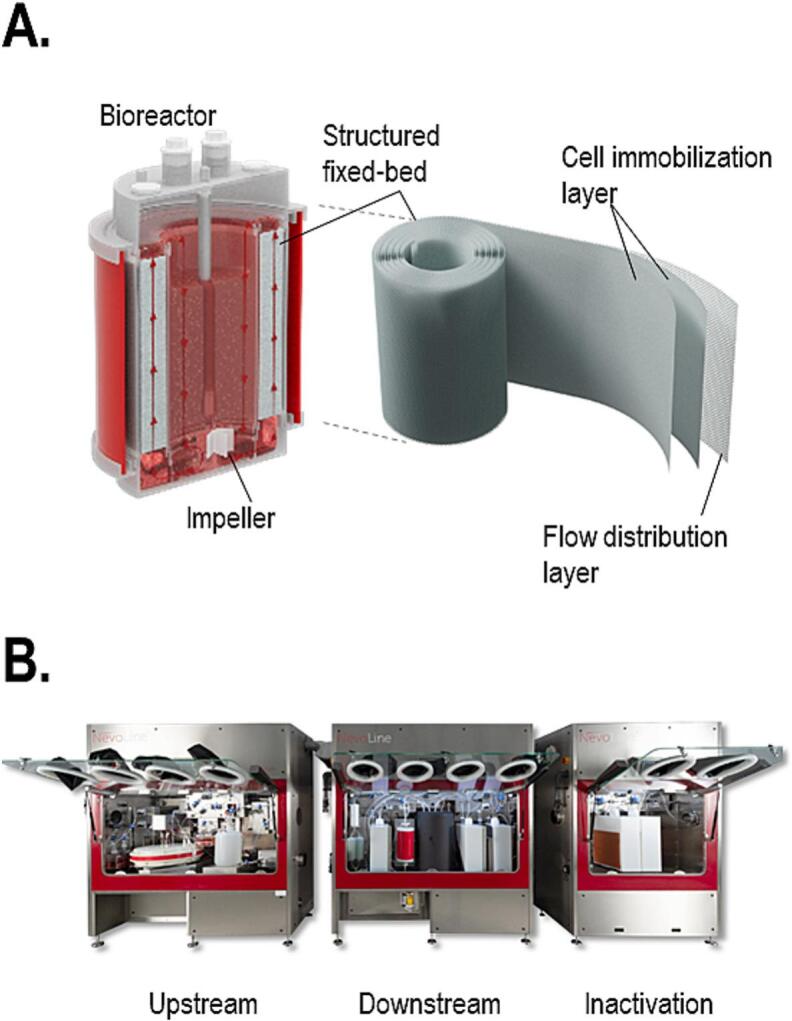
Overview of the scale-X fixed-bed bioreactor and NevoLine Polio prototype. A) The scale-X bioreactor technology uses a structured fixed-bed matrix composed of two immobilization layers, alternated with a single spacer layer for flow distribution. The fixed-bed is enclosed in a bioreactor vessel with integrated process control and recirculation using an impeller. B) The NevoLine prototype developed for the sIPV process, composed of 3 isolators respectively containing upstream, downstream and inactivation process steps.

Univercells Technologies has developed the scale-X structured fixed-bed bioreactor, which encloses a spiral-wound fixed-bed matrix composed of two immobilization layers, alternated with a single-spacer layer for proper media flow (Fig. 1A). The fixed-bed is enclosed in a bioreactor vessel with complete process monitoring and control. An impeller drives internal recirculation through the fixed bed, providing continuous perfusion of nutrients and dissolved gas to entrapped cells. The bioreactor range includes the scale-X hydro bioreactor, carbo bioreactor and nitro bioreactor models providing from 2.4 m^2^ to 600 m^2^ surface available for adherent cell growth [17]. The present study was performed using the scale-X hydro 2.4 m^2^, carbo 30 m^2^, and a prototype 150 m^2^ nitro bioreactor. At the time of performing the reported work, a 150 m^2^ prototype bioreactor, representative of the current 200 m^2^–600 m^2^ manufacturing sizes, was available.

### 2.2 Development of an integrated polio manufacturing system

The present article describes the sIPV production process, for which Univercells Technologies developed the NevoLine Polio prototype. This system was designed as 3 interconnected isolators containing equipment and single-use consumable items for upstream, downstream and inactivation process steps (Fig. 1B). The Upstream isolator operated the prototype of 150 m^2^ scale-X nitro bioreactor with a chained tangential flow filtration system, enabling a continuous collection and concentration of the virus in a perfusion process. The Downstream unit contained equipment for clarification and purification by chromatography as well as pre-inactivation. The third unit was designed for the final inactivation of the purified product. The NevoLine Polio system was designed with the goal of facilitating compliance with requirements listed in the WHO Global Action Plan (GAP) for Poliovirus Containment. WHO GAPIII requirements were current at the time of the present study and have since been updated to GAPIV. Compliance with these requirements necessitates a high level of biosecurity to prevent virus escape into the environment or via the operators [4]. Several automated safeguards were designed to support GAP compliance, with decontamination achieved through 1) in-line thermal decontamination of waste fluids; 2) thermal decontamination of internal components and single-use assemblies located in the enclosure; and 3) vaporized hydrogen peroxide decontamination of air and surfaces inside the enclosure.

### 2.3 Vero cell thawing and pre-culture before fixed-bed bioreactor culturing

For all experiments, WHO 10–87 Seed Vero cells passage 134th OCT 1987 No:0360, which had been expanded and cell banked in-house at a passage number of 142 and 3 × 10^6^ cells/vial, were used. For each experiment a cryo-vial of this Vero cell bank was thawed and expanded in T-flasks, followed by Cell Stacks (Corning), until sufficient viable cells were obtained to inoculate the bioreactor at 5000 cells/cm^2^ in the 2.4 m^2^, 30 m^2^, and 150 m^2^ scale-X fixed-bed bioreactors (Univercells Technologies).

### 2.4 Viruses

The three Sabin poliovirus research strains used in this study (sPV-1, sPV-2, sPV-3) were plasmid derived based on GenBank sequences (see Table 1). Virus seeds were generated by infecting a Vero cell culture and incubated for 3 to 4 days in a humidified incubator at 32.5 °C or 37.0 °C at 10 % CO_2_. Confluency of the virus cell culture was assessed daily and harvested on day 4 or when ≥90 % CPE was observed. The supernatant was collected and aliquoted in 1 mL cryovials and stored at ≤ − 65 °C.

**Table 1:** Virus seed characteristics.

	sPV-1	sPV-2	sPV-3
GenBank code	AY184219	AY184220	AY184221
Virus seed infectious titer [TCID_50_/mL]	1.01 × 10^9^	8.7 × 10^9^	9.33 × 10^7^

### 2.5 Virus (sPV) production in fixed-bed bioreactors

Vero cells were inoculated in the fixed-bed bioreactor (Univercells Technologies) within a seeding density range of 5000–6000 cells/cm^2^. The temperature of the cell culture was maintained at 37 °C using a sensor and heating pad, the pH was measured with an EasyFerm Plus HB pH sensor (Hamilton) and kept at 7.2 with a dead band of 0.1 through the addition of CO_2_ gas or 1 N NaOH solution. The dissolved oxygen concentration was measured with VisiFerm DO (Hamilton) and kept at 50 % through the addition of O_2_ gas. The headspace of the 30 m^2^ bioreactor was aerated with 30 mL/min air using a Mass Flow controller and the fractions of CO_2_ and O_2_ were increased based on the DO and pH.

After cell attachment, growth medium was recirculated for 5–6 days to reach a target of >150,000 cells/cm^2^. Metabolites samples were taken periodically, and (for biosafety reasons) heat inactivated for 2 h at 72 °C before analysis using the Bioprofile Flex 2 (Nova Biomedical).

After 5–6 days the cell culture media was exchanged to initiate the virus culture stage by switching from recirculation to perfusion of the fixed-bed bioreactor. The temperature and the DO were lowered to 32.5 °C to 25 %, respectively.

The cell density was determined by counting the number of cells of 3 fixed bed fiber samples and averaging to estimate the virus seed quantity needed to achieve the target MOI. After thawing cryovials of the virus seed (Table 1), the virus seed was added to the Vero cells in the bioreactor at a MOI of 0.1 and a 2 h batch-mode operation was applied to allow for infection of the cells.

### 2.6 Unstructured bioreactor (iCellis) Vero cell growth

The two main classes of fixed-bed matrix are structured and unstructured. For a structured matrix, the distribution of matrix layers throughout the domain is known. In an unstructured fixed-bed there is a random packing of the fixed-bed matrix (fibers). While in a structured fixed-bed the layers are evenly spaced throughout. To make a comparison with existing fixed-bed bioreactors, similar to structured fixed-bed bioreactor experiments, Vero cells were inoculated in the 4 m^2^ unstructured fixed-bed bioreactor (iCellis, Cytiva) at a seeding density of 5000 cells/cm^2^. Temperature, pH, and aeration were controlled similarly to the 2.4 m^2^ structured fixed-bed bioreactor.

### 2.7 Formulation of PV-3

Purified monovalent sPVs bulk material was diluted by adding of 10× M199 containing 50 g/L glycine to achieve a 1× M199 and 5 g/L glycine concentration and subsequently filtered using an Optiscale SHC 150 0.5/0.2 μm filter with 140 cm^2^ surface area (Millipore) and flux of 300 Liter per square meter hour and a max pressure of 0.8 bar(g) and stored at 2–8 °C before inactivation.

### 2.8 Inactivation

The purified bulk material was diluted with M199 medium containing 5 g/L glycine to <2400 DU/mL (PV3) and subsequently filtered with an Optiscale SHC 150 0.5/0.2 μm filter with 140 cm^2^ surface area (Millipore) and flux of 300 LMH and a max pressure of 0.8 bar (g). After filtration M199 + 1 % formaldehyde was added to a final concentration of 3.3 mM (0.01 %), filtered with a Optiscale SHC 150 0.5/0.2 μm filter and incubated for 6 days at 37 °C. After 6-days the pH was measured to ensure 7.0 ± 0.2, filtered with 0.5/0.2 μm filter and incubated at 37 °C for 7 days. After 7 days the pH was measured again and filtered with 0.5/0.2 μm filter before storage at 2–8 °C. The kinetics of inactivation were determined using daily TCID_50_ measurements and completeness of inactivation was determined by viral challenge according to Annex 2 WHO technical report 910, 2002 [18].

### 2.9 Analytical assays and methods

Infectious virus titer by TCID_50_: The 50 % Tissue Culture Infective Doses (TCID_50_) per sPV were quantified from an endpoint-dilution assay. Vero cells were cultured in a 175 cm^2^ T-Flask with MEM 10 % non-heat inactivated (nHi) FBS with 4 mM l-Glutamine and incubated at 37 °C with 5 % CO_2_ for 3 to 4 days. The attached cells were washed twice with DPBS and cells were counted with the NucleoCounter. Cells were seeded in multi-well 96 plates (TC-treated) at 5.1 × 10^3^ cells per well (100 μL) and incubated at 32.5 °C and 5 % CO_2_. Cells were infected within 4 h after seeding by the addition of 150 μL of M199 + 5 % nHi FBS (infection media) and 75 μL a serial dilution series of the sample in infection media for 8 replicates. CPE was scored 7-days post-infection. The negative control wells were verified for the absence of CPE formation.

D-antigen quantification: D-antigen units (DU) were quantified by an in-house developed sandwich ELISA method. EDQM P2160000 (Poliomyelitis vaccine (inactivated) BRP) was used as standard containing 320 DU/mL (PV-1), 78 DU/mL (PV-2) and 288 DU/mL (PV-3). Poliovirus samples were captured by strain-specific α-Polio Virus antibodies (rabbit polyclonal), which were coated in a 96-well plate (MaxiSorp C96, ThermoFisher) in 1× Phosphate-buffered saline (PBS) for at least 12 h at 4 °C. Microtiter plates were washed with 0.05 % (*v*/v) Tween-20 in 1× PBS using a plate washer to remove unbound components. Plates were blocked with 1 % (*w*/*v*) BSA and 0.05 % Tween-20 in 1× PBS for 30–90 min at 36 °C before samples were transferred into the coated plates. Samples were incubated at 36 °C for 60 to 75 min followed by a wash step of 0.05 % (v/v) Tween-20 in 1× PBS. Next, plates were incubated with detection antibodies (biotinylated strain-specific α-Polio virus antibodies, rabbit polyclonal) at 36 °C for 60 to 75 min, resulting in an immunological sandwich complex. Horseradish peroxidase labeled Extravidin was bound to the immobilized biotin, which was detected by adding the substrate TMB for 30 min at RT in the dark. The substrate reaction was stopped by the addition of the stop reagent 1 M H_2_SO_4_ and absorption was measured within 30 min at 450 nm and at 630 nm for background subtraction using a plate reader.

The tested reference dilutions form a 4 parameter-logistic curve with potency (DU/ml) on the x-axis and absorption (OD_450_-OD_630_) on the y-axis, which was used to calculate the sample potency by inverse prediction of the sample absorptions (OD_450_-OD_630_). The potencies of at least 2 valid sample dilutions were required within the range of the reference curve to calculate the average sample potency.

### 2.10 Cell density determination

To determine the cell density on the fixed-bed surface the bioreactor is equipped with several fibers of the fixed-bed material that can be pulled from the fixed-bed during the process. The attached cells on the fiber were lysed in buffer A (ChemoMetec) and the number of cells on the fibers were determined with the NucleoCounter (ChemoMetec).

Metabolite and glucose determination: Media samples were taken periodically during Vero cell expansion and viral production in the fixed-bed bioreactors, and heat inactivated for 2 h at 72 °C. Metabolites (incl. Glucose and lactate levels) were analyzed using the Bioprofile Flex 2 (Nova Biomedical).

Yield-based cell density prediction: To reduce the chances of contamination due to sampling, and to reduce the number of manual interactions with the bioreactor, a biomass glucose yield was determined from the bioreactor experiments. The observed glucose and cell density (cells/cm^2^) concentrations were used to estimate the growth rate and biomass-glucose yield. The yield and growth rate were assumed to be constant over the period (days 0 to 5). For each sampling point, the relative squared standard error was determined and summed over the period. The sum of the relative squared standard error was minimized using GRG non-linear solver in Microsoft Excel using the default parameters. The initial parameters were set from the previous study [19]. No data was excluded from the analysis.

## 3 Results

### 3.1 Development of a fixed-bed bioreactor culture process

**Fig. 2:**
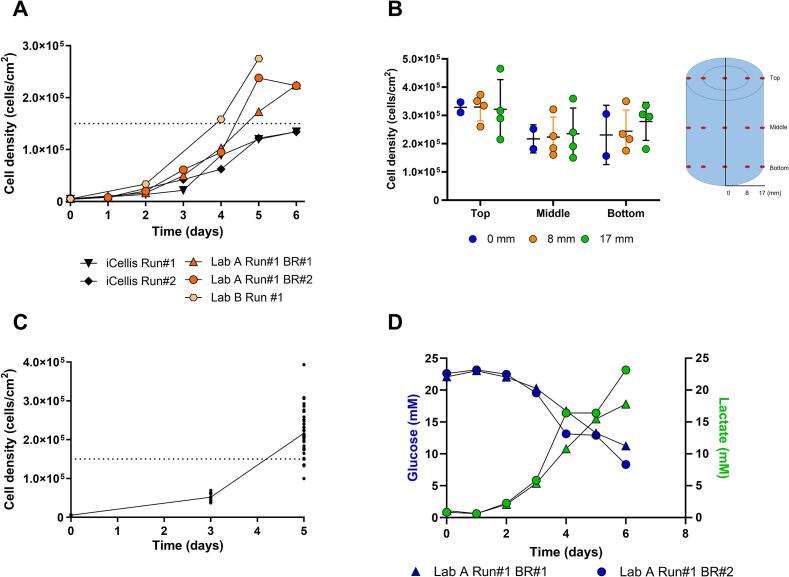
Cell densities and cell growth in fixed-bed bioreactors. (A) Comparison of the cell density of WHO Vero 10–87 cell line in three 2.4 m^**2**^ structured fixed-bed bioreactors (from 2 separate laboratories in different locations belonging to different companies) and two 4 m^**2**^ unstructured fixed-bed bioreactors over time. The cell density was determined from picked fibers of the fixed bed. The target cell density of 1.5 × 10^5^ cells/cm^**2**^ is indicated with a dotted line. (B) The structured fixed-beds of two 2.4 m^**2**^ bioreactors were dissected to analyze the distribution of cell growth across the fixed-bed surface with a radial dissection (0, 8, 17 mm) and across the height of the 10 cm fixed-bed (top, middle, bottom). Data are represented as mean ± standard deviation. (C) Vero cell density over time was assessed using fiber picking for 36 independent runs on 2.4m^**2**^ fixed-bed bioreactors. The target cell density of 1.5 × 10^5^ cells/cm^**2**^ is indicated with a dotted line. (D) Glucose (blue) and lactate (green) concentrations over time of Vero cells on the structured fixed-bed bioreactors. (For interpretation of the references to colour in this figure legend, the reader is referred to the web version of this article.)

Fixed-bed bioreactors enable the upscaling of adherent cell line cultures using a large surface area in a compact footprint. Cell growth in the developed structured fixed-bed bioreactor was compared to an existing unstructured fixed bed technology based on fiber picking of the fixed-bed and metabolite samples. A target cell density of 1.5 × 10^5^ cells/cm^2^ on day 5 was defined as sufficient to meet the intensification target of the Vero cell culture process based on previous sIPV studies [9]. Starting from a seed cell density of 5 × 10^3^ cells/cm^2^, cell densities of 2.2 ± 0.0 (× 10^5^) cells/cm^2^ were obtained on day 6 post-seeding in the structured-fixed-bed bioreactors (2.4 m^2^ culture area). In all 3 runs using the structured fixed bed, the target cell density of 1.5 × 10^5^ cells/cm^2^ was achieved by day 5. In comparison, the two unstructured fixed-bed bioreactors reached a lower cell density of 1.3 ± 0.0 (× 10^5^) cells/cm^2^ on day 6 post-seeding using the same starting density. Of note: the three runs on structured fixed beds were performed in two different laboratories as indicated **(**Fig. 2A). The low inter-operational variability observed between the runs suggests a high accuracy and robustness of the technology.

Based on these results, further experiments were performed in structured fixed-bed bioreactors only. First, the uniformity of the cell growth across the fixed bed was analyzed by determining cell density in different layers (0, 8, and 17 mm from the core) and heights (bottom, middle, and top, see Fig. 2B) of the fixed-bed bioreactor. On day 6, cell growth was uniform across the layers of the fixed-bed filter with 2.6 ± 0.6 (× 10^5^) cells/cm^2^ (0 mm), 2.7 ± 0.6 (× 10^5^) cells/cm^2^ (8 mm), 2.8 ± 0.4 (× 10^5^) cells/cm^2^ (17 mm, Fig. 2B). However, the top of the fixed bed displayed on average a higher cell density compared to the middle and bottom of the fixed bed 3.3 ± 0.0 (× 10^5^) cells/cm^2^ (top), 2.3 ± 0.1 (× 10^5^) cells/cm^2^ (middle) and 2.5 ± 0.2 (× 10^5^) cells/cm^2^ (bottom, Fig. 2B).

Repeating the runs 36 times revealed that for 31 out of 36 fixed-bed bioreactor runs at 2.4 m^2^ the desired cell density of 1.5 × 10^5^ cells/cm^2^ was reached at day 5 (Fig. 2C). Glucose and lactate analyses over time in the growth medium revealed the expected reduction of glucose and increase of lactate over time and identified day 5 as the day for medium refreshment (Fig. 2D).

### 3.2 Footprint reduction through TFF process

**Fig. 3:**
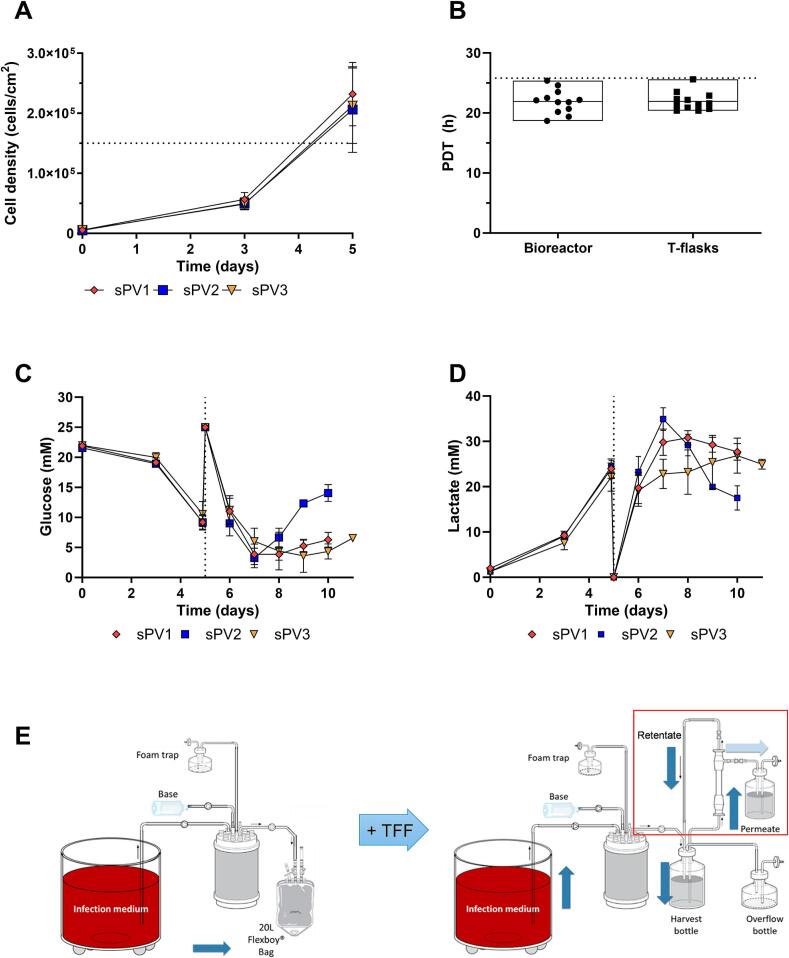
Implementation of TFF for footprint reduction of the sIPV process (*n* = 4 biological replicates per virus serotype with *n* = 3 technical replicates for fiber picking each). (A) Vero cell densities were determined through fiber picking before media replacement and infection with PV strains on day 5. The target cell density of 1.5 × 10^5^ cells/cm^**2**^ is indicated with a dotted line. (B) Population doubling times (PDTs) of Vero cells cultured in the 2.4 m^**2**^ fixed-bed bioreactor or T-flasks. Data is represented with min-to-max floating bars with the mean. To reach the target of 1.5 × 10^5^ cells/cm^**2**^ on day 5 from 6000 cells/cm^**2**^ seed a PDT of ≤25.8 h is required and depicted with a dotted line. (C & D) Glucose and lactate levels in the culture medium over time. The time of the media replacement and viral infection (day 5) is indicated with a dotted line. (E) Scheme describing the process of media replacement in perfusion mode after viral infection (infection medium), and subsequent continuous viral harvest using without (left panel) and with TFF (right panel, red box) for manufacturing footprint reduction. During the TFF, the (easily decontaminated) permeate is discharged and the harvest is concentrated in the harvest bottle. (For interpretation of the references to colour in this figure legend, the reader is referred to the web version of this article.)

Vero cell densities (determined through fiber picking) reached the predefined target of ≥1.5 × 10^5^ cells/cm^2^ by day 5 in *n* = 4 biological replicate runs per sPV serotype (*n* = 12 runs in total) dedicated for subsequent sPV 1–3 production (Fig. 3A). The equivalent population doubling time (PDT) target of ≤25.8 h was reached and was comparable to the PDT in a parallel T-flask control experiment at 21.8 ± 2.0 h versus 21.9 ± 1.5 h in the fixed-bed bioreactor (Fig. 3B). The obtained cell growth data confirms the conclusion of the independent experiment in Fig. 1 that the target of ≥1.5 × 10^5^ cells/cm^2^ can be met.

After simultaneous media replacement and infection with respectively sPVs 1–3 in separate bioreactor runs, the glucose concentration dropped severely, and lactate increased rapidly over 2 days in response to the viral infection (Fig. 3C,D). The continuous perfusion of the fixed bed with fresh medium prevented nutrient depletion (glucose levels remained at ≥1.1 mM). For sPV-2 runs, glucose consumption, and lactate production were both lower than for sPV-1 and sPV-3, indicating lowered cell death or cell metabolism for the sPV-2 infected cell culture for not investigated reasons.

Vero cells can be infected with poliovirus at a low MOI which reduces the virus seed quantity needed but results in a longer duration of the virus production phase in the bioreactor. To prevent potential virus degradation in the bioreactor the virus can be collected continuously by adding fresh media to the production bioreactor and collecting the harvest material. To reduce the footprint of such a continuous production process, a tangential flow filtration (TFF) process was developed in combination with the structured fixed-bed bioreactor whereby the TFF (100 kDa molecular weight cut-off) reduces the volume of the collected harvest and enables the production inside a containment isolator or biosafety cabinet (Fig. 3E). Vero cell growth and subsequent poliovirus (sPV) production was characterized in 2.4 m^2^ structured fixed-bed bioreactors placed in a standard biosafety cabinet. The results obtained at the 2.4 m^2^ fixed-bed bioreactor scale were used as a first indication for the anticipated D-antigen levels and virus titers to be achieved at pilot scale and manufacturing scale.

### 3.3 Scale-up of sPV production

A 30 m^2^ fixed-bed bioreactor was designed to scale-up the TFF sPV process by maintaining linear speed of the media across the fixed bed constant. The 30 m^2^ fixed-bed consisted of 3 stacks of fixed-bed of 10 m^2^ each and was operated inside the biosafety cabinet for containment reasons.

To reduce the contamination risks of cell density measurements by fiber-picking the observed cell-glucose yield was used to estimate the Vero cell densities in the fixed-bed bioreactor similar to Jiang et al. [19]. Briefly, the viable Vero cell yield on glucose and the growth rate were assumed to be constant over the cell growth period (days 0 to 5).

**Fig. 4:**
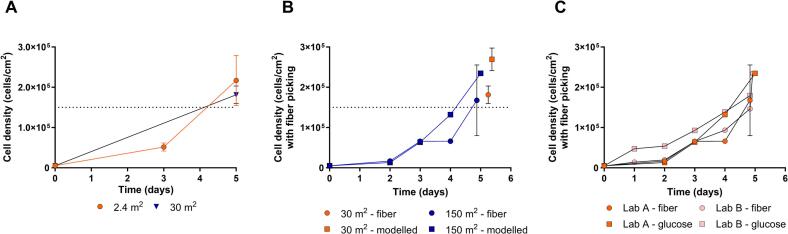
Scale-up of the TFF sPV process to 30 m^2^. (A) Vero cell density over time at 2.4 m^2^ and 30 m^2^ fixed bed measured with fiber picking (2.4 m^2^) or modeled based on the biomass glucose yield from glucose levels in the culture medium (30 m^2^). The target cell density of 1.5 × 10^5^ cells/cm^2^ is indicated with a dotted line. (B) Vero cell growth over time in 30 m^2^ and 150 m^2^ fixed-bed bioreactors was measured by fiber-picking and calculated based on medium glucose levels. Data obtained in laboratory A. The target cell density of 1.5 × 10^5^ cells/cm^2^ is indicated with a dotted line. (C) Comparison of Vero cell density over time at 150 m^2^ fixed bed, data from laboratory A vs B.

Scale-up of the 2.4 m^2^ fixed-bed TFF process to 30 m^2^ fixed-bed resulted in similar cell growth and cell densities at day 5 post-inoculation (Fig. 4A). The cell density estimated from the regression model overestimated the cell densities by approx. 0.5 × 10^5^ cells/cm^2^, when compared with the fiber picking-based cell counting. This overestimation indicated that this first version of the model needs to be further refined before being considered for use during routine sIPV production.

To accommodate large scale polio vaccine manufacturing a 150 m^2^ fixed-bed bioreactor was designed as the next scale-up step. Growth of the Vero cell line and glucose consumption in the 150 m^2^ fixed-bed bioreactor followed similar patterns after seeding when compared with that in the 30 m^2^ fixed-bed bioreactor (Fig. 4B). On day 5 post seeding the cell density on the fixed-bed surface was estimated by sampling 5 fibers of the fixed bed. The cell density on day 5 was on average comparable between the 30 and 150 m^2^ fixed-bed scales at 1.8 ± 0.2 (× 10^5^) and 1.7 ± 0.9 (× 10^5^) cells/cm^2^, respectively. In contrast to results obtained earlier for the 30 m^2^ fixed-bed bioreactors, the observed variation in the cell counts was considerably larger for the 150 m^2^ fixed-bed. However, on average the cell density target was met.

To confirm the results obtained, the Vero growth curve experiment was repeated in laboratory B using the same protocol for the 150 m^2^ fixed-bed bioreactor. The Vero cell growth and glucose consumption were confirmed to be similar to the previous experiment in laboratory A and reached on day 5 a cell density of 2.3 × 10^5^ cells/cm^2^ (Fig. 4C).

**Fig. 5:**
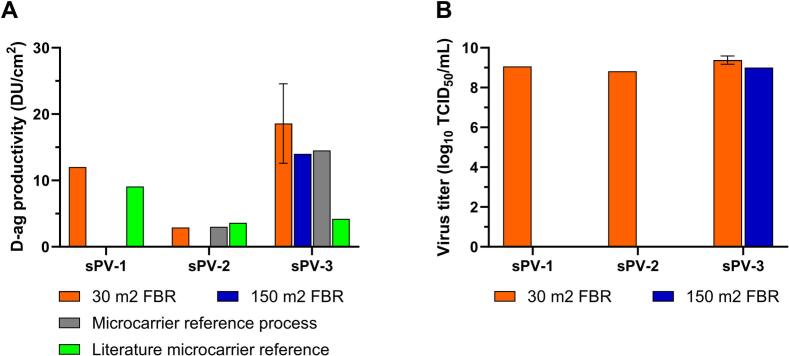
D-antigen productivity and infectious virus titers in a 30 m^2^ fixed-bed bioreactor (FBR) for three Sabin poliovirus types and proof-of-principle for scaled-up production of sPV-3 using a 150 m^2^ FBR in a containment isolator. (A) D-antigen productivity per surface area in DU/cm^2^ in the bioreactor harvest of the fixed-bed bioreactor for sPV-1 (at 30 m^2^ scale), sPV-2 (at 30 m^2^ scale) and sPV-3 (n = 3 at 30 m^2^ scale, and *n* = 1 at 150 m^2^ scale), respectively. For comparison, estimations regarding the reference process (for sPV-2 and sPV-3) and for literature data (for sPV-1, sPV-2 and sPV-3), both using 3 g/L Cytodex 1, were based on assuming the available surface area at 4400 cm^2^/g microcarriers [21]. (B) Infectious virus particles concentration in log_10_ TCID_50_/mL in the fixed-bed bioreactor harvest for sPV-1 (at 30 m^2^ scale), sPV-2 (at 30 m^2^ scale) and sPV-3 (n = 3 at 30 m^2^ scale, and n = 1 at 150 m^2^ scale), respectively. Error bars indicate standard deviation.

Next, poliovirus was produced and the sPV harvest was characterized per bioreactor scale and per sPV type. The sPV-1 D-antigen (DU) productivity per surface area (DU/cm^2^) at 30 m^2^ fixed-bed bioreactor scale was 12.0 DU/cm^2^ (Fig. 5A). While for sPV-2 and sPV-3, the D-antigen productivities at 30 m^2^ fixed-bed bioreactor scale were 2.91 DU/cm^2^ and 18.6 ± 6.0 DU/cm^2^, respectively.

For comparison of the obtained D-antigen production levels, a microcarrier-based process was run comparable to that published earlier [9] using a 10-L stirred-tank vessel with Cytodex-1 microcarriers for both sPV-2 and sPV-3. While using estimations for the available microcarrier surface area, comparable D-antigen productivities at 3.0 DU/cm^2^ and 14.5 DU/cm^2^ were observed in both bioreactor types for sPV-2 and sPV-3, respectively (Fig. 5A). When compared with microcarrier-based reference process data published earlier [20], for sPV-2 the D-antigen productivity per surface area appeared comparable (Fig. 5A). In contrast, the historical sPV-1 and sPV-3 microcarrier-based reference process D-antigen productivity data [9] appeared lower, especially for sPV-3 (Fig. 5A). However, this lower D-antigen productivity reported earlier may be attributed to use of a non-optimized microcarrier-based process especially for sPV-1 and sPV-3 [9,20], and the inevitable use of other materials, analytics, and methods in different labs. Recently it was reported Suarez-Zuluaga and colleagues that increased productivities for sPV-1 and sPV-3 were also observed after process optimization [20]. This indicated that a robust and viable USP manufacturing process was developed at both small- and pilot-scale, resulting in the decision to scale-up with a confirmation run at manufacturing scale. Also, continued process optimization and modernization may further improve the USP virus and D-antigen yields.

The infectious virus titers (log_10_ TCID_50_/mL) in the concentrated harvest from 30 m^2^ scale (Fig. 5B) were 9.06 log_10_ TCID_50_/mL, 8.82 log_10_ TCID_50_/mL, and 9.38 ± 0.2 log_10_ TCID_50_/mL for sPV-1, sPV-2, and sPV3, respectively.

### 3.4 Scale-up of Sabin Inactivated PV production to the NevoLine system

Keeping the footprint of the production site that handles the live virus as small as possible will lead to a reduced CAPEX (Capital Expenditures) for example when a new facility needs to be build or when an existing facility needs to be refurbished to meet polio GAPIII/IV requirements. To this end, a 150 m^2^ fixed-bed bioreactor was designed inside the NevoLine Polio production system, encompassing containment isolators (Fig. 1B) to scale up the sPV 30 m^2^ biosafety cabinet-based fixed-bed TFF process. Here we present the first proof-of-concept data allowing for industrial scale vaccine manufacturing using the sPV-3 production process. The NevoLine prototype encompasses the complete sIPV manufacturing process, including purification unit operations, and the inactivation step of the PV material through 0.01 % formaldehyde addition (Fig. 1B). However, for practical reasons, the presented proof-of-concept setup involved the bioreactor and TFF unit operations only.

To show feasibility for poliovirus production, the Vero cells on the 150 m^2^ fixed bed were infected with the plasmid-derived sPV-3 strain 5 days after cell seeding, and the virus was harvested 5-days post infection. The D-antigen productivity per surface area (DU/cm^2^) and infectious virus particle concentration (log_10_ TCID_50_/mL) were determined for the obtained sPV-3 harvest of one 150 m^2^ fixed-bed bioreactor. The sPV-3 D-antigen productivity per surface area of 14.5 DU/cm^2^ was comparable to that in the 30 m^2^ bioreactor at 17.1 ± 2.1 DU/cm^2^ (Fig. 5A). The infectious virus particles concentration of the concentrated sPV-3 harvest reflected the D-antigen pattern and were comparable at both scales at respectively, 9.8 for the 30 m^2^ fixed-bed bioreactor, and 9.3 log_10_ TCID_50_/mL for the 150 m^2^ fixed-bed bioreactor (Fig. 5B).

## 4 Discussion

The polio eradication program strives to switch from OPV to IPV, including sIPV. For this switch to be meaningful and applicable in the polio eradication program, lowering the cost of production of sIPV is essential. Polio vaccines have been industrialized using microcarrier technology at large scale [22], which have replaced static, and roller-bottle-based cell cultures. Microcarrier-based manufacturing provides a scale-up of the cell culture process using well-defined suspension process technology, however, this requires a large footprint as the cell density of the cell culture is usually low. In general, agitation needs to be minimized for Vero cell growth to minimize shear stress, and aeration is often only performed via the bioreactor headspace which has a poor volumetric mass transfer coefficient [12,13].

The recommendation of the WHO describes the use of the Vero or MRC-5 or cell line [23] for polio vaccine production with the WHO Vero reference cell bank 10–87 available for distribution to vaccine manufacturers. However, these cell lines recommended to produce polio vaccines are both anchorage-dependent and are therefore more challenging for scale up in comparison to suspension-based cell lines. With this paper, we provide the first evidence that the scale-X structured fixed-bed bioreactor technology can be used at different scales requiring a small footprint of manufacturing by concentrating the adherent Vero cells on a compact surface area.

When the recirculation and perfusion rate were linearly scaled up across bioreactor scales, and most prominently shown for the scale-up of 2.4 to 30 m^2^, DU concentrations per cell obtained for sPV1–3 remained stable and were comparable to the literature references [9, 20]. A first trial run of sPV-3 production at 150 m^2^ also suggests the feasibility of a linear scale-up to manufacturing size bioreactor scale. Importantly, the obtained infectious virus titers (expressed in log_10_ TCID_50_/ml) for the different bioreactor scale experiments are comparable, and highlight that our approach may be linearly scalable.

Characterizing the manufacturing process, we showed that sufficient Vero cell growth to 1.5 × 10^5^ cells/cm^2^ at day 5 of expansion can be reached irrespective of the fixed-bed bioreactor scale used. This cut-off was defined as sufficient to meet the intensification target of the cell culture process based on previous sIPV studies [9]. Also, the resulting product recoveries based on DU-antigen units from our tested bioreactor scales show comparable productivity per cell when compared to the reference process [9].

For other vaccines, small-scale unstructured fixed-bed experiments showed promising results in producing Rabies, hepatitis-A, and Chikungunya vaccines [24]. Comparison of the growth data between an unstructured and structured fixed bed in this study showed improved Vero cell density in the tested structured fixed bed compared to that in unstructured counterparts. Supporting this data, the extent of cell growth in our experience is in line and even extends earlier published Vero cell growth in a similarly sized (10 m^2^) previous version of the fixed-bed bioreactor from the same manufacturer (Univercells Technologies) [25].

Cell growth was uniform across the fixed bed at the 2.4 m^2^ scale using our perfusion and culturing protocol, which is challenging at high cell density, as described by others [26]. Though the growth uniformity was not tested for larger fixed-bed bioreactor scales, the growth uniformity is expected to decrease with increasing bioreactor sizes due to e.g., gravity and nutrient shifting. This distribution-disturbing effect was counteracted in our fixed-bed bioreactors by automated recirculation and perfusion of the culture medium. However, as evidenced by the comparable DU productivity across scales, a linear scale-up of these culturing and recirculation/perfusion parameters for larger bioreactor sizes seems sufficient for the scale-up of the manufacturing process.

We furthermore showed that TFF can be applied within an integrated continuous process. The advantage of TFF over normal flow filtration is that most TFF devices are linearly scalable (and reusable after cleaning) and thereby significantly reduce consumable costs of this production process step [27,28].

Sampling of adherent cell cultures is an inherent problem for the control and monitoring of the process. The scale-X bioreactors allow for fiber picking of fixed-bed material to determine the cell surface area density, however this is not a preferred method due to contamination risks associated with the opening of the bioreactor. Progression of the cell culture can be inferred by glucose consumption which has previously been modeled for Vero cells for micro-carrier technology [19]. In our hands, the developed model slightly overestimated Vero cell growth when compared to fiber picking, particularly in the larger fixed-bed bioreactor scales.

A somewhat increased cell-glucose yield of approx. 86 × 10^6^ cells/mmol glucose was estimated after 5 days of growth culture in the 150 m^2^ fixed bed when compared to the average 51 × 10^6^ cells/mmol glucose reported for microcarrier-based batch cultures [19]. However, the lactate concentrations in the culture medium before infection at day 5 reached up to 23 mM during the growth phase similar to that reported by Petiot et al. [29]. This high acidification may highlight the potential spatial oxygen limitation of the Vero cell culture within the fixed bed, as also described earlier [30]. Nonetheless, also for the larger scales (30 and 150 m^2^), the desired total cell density of ≥1.5 × 10^5^ cells/cm^2^ was reached at day 5, and linear scale-up of sIPV manufacturing based on D-antigen units was observed.

While experimentally identifying the scale-up parameters of the fixed-bed bioreactors is beyond the scope of this study, at least a hypothesis can be formulated of critical process parameters. Linear scale-up of sIPV manufacturing based on D-antigen units was observed when the recirculation and perfusion rate were maintained across bioreactor scales. Furthermore, the present study demonstrated the ability to transfer the scale-X fixed-bed bioreactor sIPV manufacturing process into the NevoLine integrated isolator prototype. This represents a successful proof for the feasibility of producing sIPV vaccine within a self-contained microfacility in facilitating compliance to WHO polio containment requirements.

## 5 Conclusions

With this study, we show for the first time that poliovirus vaccine production at a low-footprint container-sized (isolator) production site is feasible and that the use of structured fixed-bed bioreactors may enable linear upscaling of Vero cell growth and virus production. Subsequent TFF-mediated harvest and downstream processing allow virus production at virus titers and DU-antigen concentrations that align with published microcarrier-based production levels.

The reported results, which were generated with Vero cells under a limited set of experimental conditions, can be used as a starting point for future research into the cultivation and optimization of anchorage-dependent cell lines in high cell densities thereby improving viral vaccine output in a small footprint [26].

## CRediT authorship contribution statement

**Ahd Hamidi:** Writing – review & editing, Visualization, Supervision, Project administration, Methodology, Funding acquisition, Conceptualization. **Marieke Willemsen:** Supervision, Project administration, Formal analysis. **Thomas Robert:** Writing – review & editing, Writing – original draft, Visualization, Software, Resources, Investigation, Conceptualization. **Jean-Christophe Drugmand:** Visualization, Software, Resources, Investigation. **Mónika Z. Ballmann:** Validation, Supervision, Methodology, Formal analysis. **Pim Velthof:** Investigation. **Hans Verdurmen:** Investigation. **Ana Catarina Pinto:** Validation, Formal analysis. **Jochem Pronk:** Investigation. **Laura Palladino:** Investigation. **Menzo Havenga:** Writing – review & editing, Writing – original draft, Visualization. **Chris Yallop:** Supervision, Project administration, Funding acquisition, Conceptualization. **Wilfried A.M. Bakker:** Writing – review & editing, Writing – original draft, Visualization, Methodology, Data curation.

## Financial support

Financial support for this work was received from The Bill and Melinda Gates Foundation under BMGF, grant numbers: INV-008552 (OPP1154686), OPP1203429, and INV-020859, which did not influence the outcomes of the research. All authors confirm to have contributed to the manuscript, and that the manuscript has been read and approved for submission by all the named authors.

## Transfer of copyrights

The copyright of the manuscript will not be transferred to the journal publisher upon acceptance for publication. This work was supported, in whole or in part, by the Bill & Melinda Gates Foundation Grant Numbers: INV-008552 (OPP1154686), OPP1203429, and INV-020859. Under the grant conditions of the Foundation, a Creative Commons Attribution 4.0 Generic License has already been assigned to the Author Accepted Manuscript version that might arise from this submission. Batavia Biosciences and Univercells Technologies retain the rights to use the underlying information included in this manuscript.

## Declaration of competing interest

The authors declare that they have no known competing financial interests or personal relationships that could have appeared to influence the work reported in this paper.

## Data Availability

Data will be made available on request.
